# Influence of research evidence on the use of cardiovascular clinical prediction rules in primary care: an exploratory qualitative interview study

**DOI:** 10.1186/s12875-023-02155-w

**Published:** 2023-09-20

**Authors:** Jong- Wook Ban, Rafael Perera, Veronika Williams

**Affiliations:** 1 Centre for Evidence-Based Medicine, University of Oxford, Oxford, UK; 2 Department for Continuing Education, University of Oxford, Oxford, UK; 3 Nuffield Department of Primary Care Health Sciences, University of Oxford, Oxford, UK; 4 School of Nursing, Nipissing University, North Bay, ON Canada

**Keywords:** Clinical prediction rule, Research evidence, Primary care, Evidence-based medicine

## Abstract

**Background:**

Cardiovascular clinical prediction rules (CPRs) are widely used in primary care. They accumulate research evidence through derivation, external validation, and impact studies. However, existing knowledge about the influence of research evidence on the use of CPRs is limited. Therefore, we explored how primary care clinicians’ perceptions of and experiences with research influence their use of cardiovascular CPRs.

**Methods:**

We conducted an exploratory qualitative interview study with thematic analysis. Primary care clinicians were recruited from the WWAMI (Washington, Wyoming, Alaska, Montana and Idaho) region Practice and Research Network (WPRN). We used purposeful sampling to ensure maximum variation within the participant group. Data were collected by conducting semi-structured online interviews. We analyzed data using inductive thematic analysis to identify commonalities and differences within themes.

**Results:**

Of 29 primary care clinicians who completed the questionnaire, 15 participated in the interview. We identified two main themes relating to the influence of clinicians’ perceptions of and experiences with cardiovascular CPR research on their decisions about using cardiovascular CPRs: “Seek and judge” and “be acquainted and assume.” When clinicians are familiar with, trust, and feel confident in using research evidence, they might actively search and assess the evidence, which may then influence their decisions about using cardiovascular CPRs. However, clinicians, who are unfamiliar with, distrust, or find it challenging to use research evidence, might be passively acquainted with evidence but do not make their own judgment on the trustworthiness of such evidence. Therefore, these clinicians might not rely on research evidence when making decisions about using cardiovascular CPRs.

**Conclusions:**

Clinicians’ perceptions and experiences could influence how they use research evidence in decisions about using cardiovascular CPRs. This implies, when promoting evidence-based decisions, it might be useful to target clinicians’ unfamiliarity, distrust, and challenges regarding the use of research evidence rather than focusing only on their knowledge and skills. Further, because clinicians often rely on evidence-unrelated factors, guideline developers and policymakers should recommend cardiovascular CPRs supported by high-quality evidence.

**Supplementary Information:**

The online version contains supplementary material available at 10.1186/s12875-023-02155-w.

## Background

Health research is valuable when it creates knowledge that can improve the decisions of patients, clinicians, and policymakers; or when other researchers can use the results to synthesize such knowledge [1]. Unfortunately, many studies in health research fail to create such knowledge useful to clinical practice or policy decisions [2–4] due to shortcomings in planning, design and conduct, publication, and reporting; therefore represent research waste [4–9].

Clinical prediction rules (CPRs) are tools that estimate the likelihood of a health outcome using information from health history, physical examination, and test results of a person [10–13]. CPRs enable clinicians to incorporate multiple factors in making healthcare decisions empirically rather than intuitively [14], and can help implement evidence-based medicine (EBM) in practice [12, 15]. A CPR can be categorised as diagnostic CPR when it assesses how likely a person has a health condition currently and prognostic CPR when it evaluates how likely it is for an individual to develop a health outcome in the future [11, 16]. These CPRs can have significant impact in clinicians’ diagnostic or therapeutic decisions. For example, clinicians evaluating patients suspected of having an acute pulmonary embolism, can use the Wells rule [17, 18] to identify patients with high risk for having pulmonary embolism and increase the diagnostic efficiency [19–22] and reduce unnecessary imaging and radiation exposure [21, 23, 24], while maintaining the safety of patients [19]. Although many CPRs in a wide range of clinical domains have been developed [25], CPRs for cardiovascular conditions are most widely recognized and used in primary care [12, 26]. These cardiovascular CPRs are recommended by major guidelines [27–29] and clinicians often use these CPRs to communicate cardiovascular disease risk to patients and decide whether to start an intervention [26], such as the HMG-CoA reductase inhibitor.

Clearly justified CPRs can accumulate high-quality evidence through well designed and conducted derivation, external validation, and impact studies by demonstrating their abilities to predict an outcome accurately, performances in different populations, and potentials to improve patient care [10, 30, 31]. But for evidence accumulated through these stages of CPR development to be valuable, it should also contribute to the healthcare decisions. In other words, even high-quality research evidence about CPRs can still be research waste unless patients, clinicians, or policymakers use the evidence and improve their decisions.

Unfortunately, there is limited existing knowledge about whether and how research evidence influences the use of CPRs. Previous studies described that the presence of research evidence about CPRs [32, 33], in particular for accuracy [33–36], generalizability [34, 37–39], and effectiveness [36], influenced the use of CPRs in practice. However, findings from these studies primarily related to whether having sufficient evidence affected the use of CPRs, and more substantive knowledge about these questions is still absent.

Therefore, we conducted a qualitative interview study to examine how primary care clinicians’ perceptions of and experiences with cardiovascular CPR research influence their perceived use of cardiovascular CPR in practice. In preparation to accomplish this primary objective, we explored how primary care clinicians perceive cardiovascular CPR research and what they experience in using such research evidence as part of their practice.

## Methods

All methods were performed in accordance with the relevant guidelines and regulations of the Central University Research Ethics Committee (CUREC), University of Oxford. We conducted an exploratory qualitative interview study in accordance with an inductive thematic analysis approach suggested by Braun and Clarke [40]. We also reported our conduct and findings following the relevant reporting guidelines for qualitative interview studies [41, 42].

### Participant selection

We recruited participants from the members of the Washington, Wyoming, Alaska, Montana, and Idaho (WWAMI) region Practice and Research Network (WPRN). The WRPN is a network of diverse primary care practices located in urban, suburban, and rural areas across the five-state WWAMI region in academic, private, community, and public settings [43]. One of the functions of the WPRN is to link researchers with primary care practices for research participation and provide support [43].

Based on the numbers of participants included in existing exploratory qualitative interview studies conducted in primary care [44–46], we aimed to recruit up to 15 primary care clinicians. This goal was also set considering available resources and empirical evidence on data saturation in thematic analysis [47]. We recruited clinicians who were (1) 18 years or older, (2) willing to give informed consent, and (3) currently practicing primary care medicine such as Family Practice, Internal Medicine, Geriatrics, or General Practice in the USA. The WPRN sent e-mails with recruitment materials to the members of the WPRN Survey Research Panel, which is a registry of the WPRN members who agreed to receive electronic surveys facilitated by the WPRN Coordinating Center, inviting them to take part in the study. The members were asked to complete a brief online questionnaire to describe their use of cardiovascular CPRs and provide demographic, practice, and contact information. Also, they were asked to express their interest in participating in the interview.

We sent an invitation to schedule an interview to selected participants based on a sampling frame aimed at maximizing diversities in the cardiovascular CPR use, research experience, and research evidence consideration in decisions about using cardiovascular CPRs. We stopped recruiting additional primary care clinicians when we determined that thematic saturation was achieved after analyzing data from 15 interviews with participants who responded to the invitation.

### Data collection

One author (JWB) collected data by conducting qualitative semi-structured online interviews between 3 August 2020 and 10 September 2020. The Medical Sciences Interdivisional Research Ethics Committee (MS IDREC) of the University of Oxford conducted an ethical review and approved the study (Ethics Approval Reference: R69695/RE001). All participants consented to participate in the interview: either by sending a completed and signed written informed consent form via post or e-mail in advance, or by giving audio-recorded verbal informed consent immediately before starting the interview.

Data were collected through in-depth semi-structured interviews using an online platform (Microsoft Teams). By loosely following a pilot-tested topic guide but maintaining the natural flow of conversation, participants were asked about their use of cardiovascular CPRs, reasons for choosing the CPRs, views on research evidence regarding cardiovascular CPRs, experiences with such research evidence, and research evidence’s role in their decisions about using cardiovascular CPRs. The topic guide is included in Additional file 2. We provided a $5.00 Amazon eGift Card to clinicians who completed the online questionnaire and a $50.00 Amazon eGift Card to those who took part in the interview.

### Data management and analysis

All interviews were digitally recorded and transcribed verbatim using online software [48]. Whenever possible, information that could directly discern an individual participant was removed from transcripts. Data were securely stored in a dedicated folder within the University of Oxford's cloud service according to the University’s data storage policy.

We conducted an inductive thematic analysis using methods described by Braun and Clarke to identify semantic themes [40]. Using NVivo 12 to manage and organise data, a single coder (JWB) created initial codes by finding small data segments that might be meaningful, focusing on but not necessarily restricted to the specific research questions. Another author (VW) reviewed these codes to ensure consistency of coding framework and identify anything that might have been missed in the coding process. The researchers were reflexive of their coding approach throughout by documenting coding decisions and initial analytical thoughts using a research journal. After coding all interview transcripts, codes were categorized into themes, while reviewing data within each theme and revising codes for consistency. At the same time, using evolving concept maps as a guide, themes were reorganized by examining the relationships between the themes. This process was continued until no further significant revisions to the themes were made.

## Results

### Participants

The characteristics of primary care clinicians who completed the online questionnaire are presented in Table 1.

**Table 1:** Characteristics of primary care clinicians who completed the online questionnaire

	Interviewed, *n* = 15	Not interviewed, *n* = 14	All, *n* = 29
*Speciality*			
Family medicine	10 (66.7)	10 (71.4)	20 (69.0)
Internal medicine	5 (33.3)	4 (28.6)	9 (31.0)
*Gender*			
Female	9 (60.0)	9 (64.3)	18 (62.1)
Male	6 (40.0)	5(35.7)	11 (37.9)
*The year started practicing, median (IQR)*	2007 (1991–2017)	2004 (1995–2019)	2007 (1995–2018)
*Practice size, median (IQR)*	30 (7–48)	27.5 (10–40)	30 (10–40)
*Clinical role*			
Physician	12 (80.0)	12 (85.7)	24 (82.8)
Resident	2 (13.3)	2 (14.3)	4 (13.8)
Physician assistant	1 (6.7)	0 (0.0)	1 (3.4)
*Teaching or research experience*			
Both research and teaching	5 (33.3)	8 (57.1)	13 (44.8)
Research only	0 (0.0)	1 (7.1)	1 (3.4)
Teaching only	8 (53.3)	5 (35.7)	13 (44.8)
Neither	2 (13.3)	0 (0.0)	2 (6.9)
*Practice setting*			
Urban	6 (40.0)	10 (71.4)	16 (55.2)
Suburban	6 (40.0)	2 (14.3)	8 (27.6)
Semi-rural	2 (13.3)	2 (14.3)	4 (13.8)
Rural	1 (6.7)	0 (0.0)	1 (3.4)
*Practice location*			
Western Washington	8 (55.3)	8 (57.1)	16 (55.2)
Eastern Washington	4 (26.7)	3 (21.4)	7 (24.1)
Idaho	3 (20.0)	1 (7.1)	4 (13.8)
Montana	0 (0.0)	1 (7.1)	1 (3.4)
Wyoming	0 (0.0)	1 (7.1)	1 (3.4)
*Integration of CPR in practice software*			
At least one cardiovascular CPR	9 (60.0)	5 (35.7)	14 (48.3)
No cardiovascular CPR	6 (40.0)	9 (64.3)	15 (51.7)
*Use of cardiovascular CPR in practice*			
In most or all cases	6 (40.0)	5 (35.7)	11 (37.9)
Occasionally	6 (40.0)	7 (50.0)	13 (44.8)
Rarely	3 (20.0)	1 (7.1)	4 (13.8)
Never	0 (0.0)	1 (7.1)	1 (3.4)
*Consideration of evidence when choosing cardiovascular CPR*			
Considered evidence	14 (93.3)	11 (78.6)	25 (86.2)
Did not consider evidence	1 (6.7)	3 (21.4)	4 (13.8)

Values are the number (%) of primary care clinicians unless indicated otherwise

*IQR* Interquartile range, *CPR* Clinical prediction rule

Of 29 primary care clinicians who completed the questionnaire, 15 were purposefully selected for interview according to the sampling frame presented in Table 2. The interviewed clinicians were in various career stages, between second-year resident in training and semiretired, practicing in Washington and Idaho. Their characteristics were similar to those who were not interviewed and reflect current primary care clinicians in the geographic context.

**Table 2:** Sampling frame for selecting primary care clinicians for interview

Use of cardiovascular clinical prediction rule (CPR)	Has research experience		Has no research experience	
	Considered evidence when choosing CPR	Did not consider evidence when choosing CPR	Considered evidence when choosing CPR	Did not consider evidence when choosing CPR
In most or all relevant cases	2 (4)	0 (1)	3 (4)	1 (2)
Occasionally	3 (7)	0 (1)	3 (5)	0 (0)
Rarely or never	0 (1)	0 (0)	3 (4)	0 (0)

Values are the number of primary care clinicians (values in brackets are the number of clinicians who completed the online questionnaire)

### Main themes

We identified two main themes regarding how primary care clinicians’ perceptions and experiences in relation to cardiovascular CPR research might influence the way they interact with research evidence and make decisions about using cardiovascular CPRs: “Seek and Judge” and “Be acquainted and Assume.”

### Theme 1: Seek and judge

**Fig. 1:**
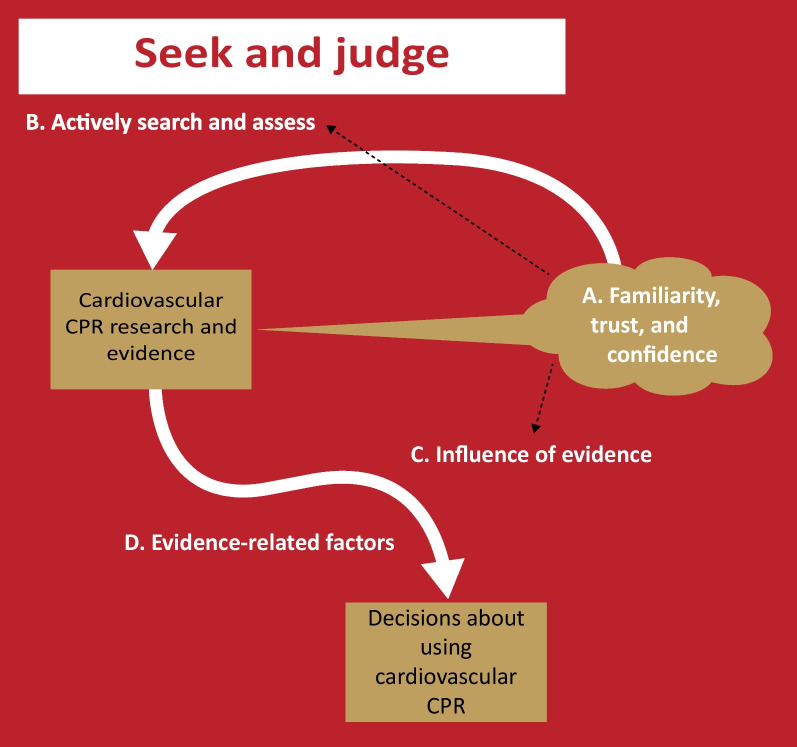
A concept map of the theme "Seek and judge." CPR: clinical prediction rule

This main theme includes four subthemes as illustrated in Fig. 1: (A) familiariry, trust, and confidence, (B) seek and judge, (C) influence of evidence, and (D) evidence-related factors. The first subtheme, “familiarity, trust, and confidence,” represents primary care clinicians’ perceptions of and experiences with cardiovascular CPR research. The second and third subthemes, “actively search and assess” and “influence of evidence,” explain how their perceptions and experiences might affect the use of research evidence. Finally, the last subtheme, “evidence-related factors,” describes some aspects of research evidence that might be important for their decisions about using cardiovascular CPRs.

A. Familiarity, trust, and confidence

Some of the primary care clinicians’ perceptions of and experiences with research might help them include research evidence in their decisions about using cardiovascular CPRs. When clinicians feel they are familiar with cardiovascular CPR research or are aware of research evidence about a cardiovascular CPR, their decisions about using the cardiovascular CPR might involve the research evidence. Participants indicated they were familiar with cardiovascular CPR research and knew some research evidence behind the cardiovascular CPRs they were using. For example, a General Internist, who learned about various cardiovascular CPRs such as CHA_2_DS_2_-VASc score [49] and Wells scores [18, 50] during residency training, had some knowledge about what cardiovascular CPR research was:

*“I think it's a very broad category, … encompasses this whole ‘how do you validate these things?’ ‘how do you use them?’ ‘are people using them accurately?’ …” (IS78)*

Also, the Internist described the evidence behind some of the cardiovascular CPRs:

*“Some of these [prediction rules] do have cost-benefit analyses that show … there are cost benefits to using these clinical [prediction rules] in the right setting.” (IS78)*

Similarly, when clinicians trust and value the research evidence, their decisions on using cardiovascular CPRs might be influenced by research evidence. Some interviewed clinicians conveyed that they trusted and had a favourable opinion of cardiovascular CPR research and the evidence it generated. A Family Physician, who used the ACC/AHA pooled cohort equation [51] routinely, explained the reason for trusting evidence around the equation:

*“I’m pretty sceptical [about] pharma’s involvement in things … [But] I think, … for example… ASCVD [equation] …, just instinctively …, there isn't like a ton of possible corruption … With like ASCVD [equation], it doesn't seem to play as much, which is reassuring.” (BG35)*

Further, when clinicians feel confident in using research evidence, their decisions on using cardiovascular CPRs might be influenced by the research evidence. Some participants, especially younger and newly trained physicians, expressed their confidence in searching for, reading, and appraising primary studies about cardiovascular CPRs. For example, an Internal Medicine resident, who rarely used the ACC/AHA pooled cohort equation [51], felt confident about searching for articles using PubMed:

*“I tend to use PubMed … I think PubMed is pretty easy. There's also a section of PubMed that only looks at clinical studies … rather than … benchtop type research. So that tends to be helpful.” (FA84)*

Similarly, another Internal Medicine resident, who used various guideline-recommended cardiovascular CPRs, felt comfortable reading and interpreting primary studies:

*“My comfort level with reading primary literature is fairly good. I feel like I had pretty decent training in like Biostatistics and [have] ability to … read primary literature and interpret what they mean when they use sensitivity, specificity, specificity, [and] number needed to treat.” (SY54)*

These clinicians who expressed confidence in using research evidence tend to be recent graduates from training or physicians in training with less than ten years of experience. They often indicated gaining their knowledge and skills during medical school and residency training through formal curricula on EBM (e.g. journal club) or informal teachings such as discussions with senior clinicians and colleagues.

B. Actively search and assess

When clinicians have favourable perceptions of or experiences with research, they might actively search and evaluate research evidence about cardiovascular CPRs. Some clinicians, who were familiar with, trusted, and felt confident in using research evidence, actively sought primary research evidence about cardiovascular CPRs using electronic databases, libraries, and online resources. For instance, a General Internist, who felt to be experienced with searching research evidence, recounted looking up a study about the PERC rule [52] while rounding with residents:

*“Residents were … using the PE rule criteria in a very high-risk patient. … So the question was, … ‘In high-risk patients, does the PE rule criteria apply?’ … I usually use a MeSH heading on PubMed to … look for [studies]. And … that was one … that had just come out in the last four to five years. And, so we pulled it in.” (GO38)*

Some clinicians who were familiar with, trusted, and felt confident in using research evidence, questioned others’ assessments of evidence and tried to assess cardiovascular CPR studies' trustworthiness independently. Some found evaluating research evidence about cardiovascular CPRs easy and valuable but others felt lost and disappointed when they did not understand the information. A General Internist, who picked up searching and appraising skills during residency training, shared an expereince with cardiovascular CPR studies:

*“I … go through their methodology and … do a literature appraisal to see if it's appropriate to the clinical question I have ... Some papers are real easier to read and understand than others.” (IS78)*

But, when judging the trustworthiness of evidence, clinicians often relied on certain study characteristics and results such as sample size, population, setting, outcome, funding source, or *p*-value rather than study design and internal validity. A newly trained Family Physician, who had limited time reading research articles, discussed factors for determining trustworthiness.

*“Oh, I look at… the number of participants, … what the patient population was made up of, if it's similar to my patient population, … and then what the … the confidence interval is.” (PR88)*

An Internist was especially concenred about funding sources.

*“I look at … the size of the study, location and … multi-center. … I … look at who paid for it. … If it was being paid for by an imaging company or by the anticoagulation … company, really kind of puts my antennas on edge to the question how the statistics are going to be explained.” (GO30)*

Although some clinicians seem to actively seek and judge the research evidence about cardiovascular CPRs, their searches and appraisals may not necessarily have sufficient thoroughness and rigour according to the classic definition of EBM [53].

C. Influence of evidence

When clinicians have positive perceptions of and experiences with research, their decisions about using cardiovascular CPRs might be influenced by research evidence. Some interview participants, who were familiar with, trusted, and felt confident in using research evidence, argued that clinicians should use research evidence when making decisions about using cardiovascular CPRs and indicated that reviewing research evidence influenced whether or how they used cardiovascular CPRs. For example, a General Internist, who was experienced in reading CPR studies, argued it was important to understand the evidence behind cardiovascular CPRs before using them:

*“It is easy to just plug the numbers into a rule, um, and … make snap judgments … Whether it's preventing stroke or preventing an MI, um I think we really have to know the data before throwing medications and interventions at our patients.” (GO38)*

Further, the Internist indicated that reading articles about the TIMI and Heart scores [54, 55] led to a change in the way these cardiovascular CPRs were used in patients with chest pain:

*“It used to be anytime … someone had chest pain, I would use a TIMI. … Now, I really try to refine, … ‘is this the right person?’ or ‘is this someone … I think the HEART score makes more sense’ … you know, based on some of those initial studies.” (GO38)*

Similarly, after reviewing an article about a new cardiovascular CPR, a Family Physician who was familiar with cardiovascular CPR research felt the evidence about the new CPR was “compelling” and recommended residents to use the CPR:

*“I found it compelling. Yes, I found … it will be worth to change ... to the new numbers that they proposed compared to the older one. ... I have banked on those articles [and] decided to use those. … I tried to teach it to the residents also. … I asked all of them to download the ASCVD app to their phone. (FX91)*

Therefore, having familiarity, trust, and confidence regarding the use of research evidence might promote the involvement of research evidence in clinicians’ decisions about using cardiovascular CPRs.

D. Evidence-related factors

When clinicians’ decision about using a cardiovascular CPR involves research evidence, certain factors related to evidence might play an important role in their decisions. Clinicians who took part in this study considered three evidence-related factors: having evidence behind the CPR, evidence of applicability, and features of evidence.

To some clinicians, having evidence of accuracy, generalizability, and effectiveness was important. For example, an experienced Family Physician taught residents to use cardiovascular CPRs that had been evaluated in an external validation study:

*“It's really nice to see a validation study in a different setting, before rolling out anywhere else. … I've taught … them that most prediction rules have never been validated. So ‘you better be really sure you're using one that's at least validated!’…” (GR91)*

However, the most important factor related to research evidence that influenced the participants’ decisions about using cardiovascular CPRs, was the evidence of applicability to their own patients. Clinicians assessed whether the populations and settings of CPR studies were sufficiently similar to their patients and settings. They felt confident when applying CPRs evaluated in populations and environments similar to theirs but were reluctant to use CPRs only studied in different populations or settings. A Internal Medicine resident, who believed clinicians should consider research evidence when choosing cardiovascular CPRs, explained the reason for deciding to use the ACC/AHA pooled cohort equation [51]:

*“I tried to look at my patient population on my outpatient panel and I tried to select one that was … going to envelop my patients most appropriately.” (FA84)*

Similarly, a Family Physician argued it was important to know that the research evidence applied to patients when choosing a cardiovascular CPR:

*“I think, a good doctor … would know … if something didn't apply to your population, then … you can't extrapolate them … So, … when you're looking at studies, … ‘does it apply to your patient population?’. … That's probably a fairly early decision in using that tool.” (IN63)*

To some clinicians, certain features of research evidence such as having a large sample size, higher power, extended follow-up, multiple locations, and statistically significant results were important. A General Internist, who changed the way cardiovascular CPRs were used in practice after critically appraising primary studies, discussed some of the features of research evidence that was important:

*“For me, the biggest thing would be the sample size. If … the sample size is super small, … [I] wouldn't … use those tools. [If] studies are underpowered, … [or] the p-value was not … appropriate, I probably wouldn't use those tools as well.” (IS78)*

Likewise, having a large enough sample size was important to this Family Physician.

*“I think based on a large enough population, … I the size of the of the population data base …, statistical significance …” (KJ34)*

### Theme 2: Be acquainted and assume

**Fig. 2:**
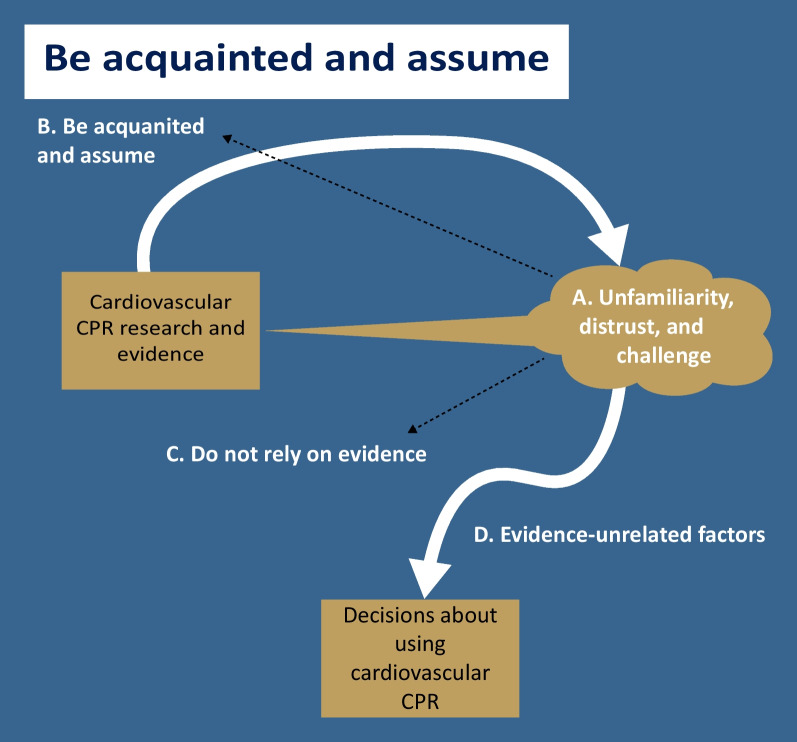
A concept map of the theme "Be acquainted and assume." CPR: clinical prediction rule

This theme also consists of four subthemes, as presented in Fig. 2: (A) unfamiliarity, distrust, and challenge, (B) be acquainted and assume, (C) do not rely on evidence, and (D) evidence-unrated factors. The first subtheme, “unfamiliarity, distrust, and challenge,” characterizes clinicians’ perceptions of and experiences with cardiovascular CPR research. The second and third subthemes, “be acquainted and assume” and “do not rely on evidence,” describe how these perceptions and experiences might influence clinicians’ interactions with research evidence. Finally, the last subtheme, “evidence-unrated factors,” explains how clinicians might make decisions about using cardiovascular CPRs when they do not rely on research evidence.

A. Unfamiliarity, distrust, and challenge

Some of the clinicians’ perceptions of or experiences with research might impede involving research evidence in their decisions about using cardiovascular CPR. For example, when clinicians are unfamiliar with cardiovascular CPR research or unaware of research evidence about a cardiovascular CPR, it would be implausible for research evidence to directly influence whether or how they use the cardiovascular CPR. In fact, some interview participants did not know any research evidence about cardiovascular CPRs that they were using or could not recall any specifics about the research evidence they might have encountered. A Internal Medicine resident who frequently used various guideline-recommended cardiovascular CPRs, including the CHA_2_DS_2_-VASc and HAS-BLED scores [49, 56], admitted having limited knowledge about the evidence behind these CPRs:

*“I may have knowledge about ... the scores, but I have limited knowledge about necessarily the research that goes into them because I can't say that I've done like a lot of digging behind them.” (SY54)*

Similarly, when clinicians distrust research evidence about a cardiovascular CPR, it could have limited influence on their decisions about whether or how they use the cardiovascular CPR. Some interviewed clinicians were sceptical about research evidence in general and shared the same views on cardiovascular CPR research. For example, a Family Physician, who had postgraduate degrees, read a paper about the CHA_2_DS_2_-VASc score [49], multiple times because of disbelieving it:

*“I had to read like three times. When I was … in my graduate school, they were all talking about hormone replacement therapy (HRT). … ‘Oh, this is the panacea, everybody should be taking HRTs.’ So, …I don't believe everything people say … I have seen so many things changing … in medicine … So, I usually read things … again … because sometimes I don't believe it.” (FX91)*

In addition, when clinicians had a difficult experience with cardiovascular CPR research or viewed using research evidence as challenging, it might have limited influence on their decisions about using cardiovascular CPRs. Common challenges in using research evidence about cardiovascular CPRs among interview participants related to lack of time, ability, and resources. Clinicians expressed difficulties using research evidence because they had busy clinical schedules and little time for reading. An academic Family Physician discussed the challenge in using research evidence due to lack of time:

*“I just think people are busy. You know, even for me as an academic, I don't have the time. I haven't made the time to go back and look at the original data and research under them. So, I think that's really hard for practising physicians to do that.” (KJ34)*

Several interview participants, especially those older physicians with more clinical, research, and teaching experiences, felt it was challenging to use research evidence about cardiovascular CPRs. Despite these experiences, they expressed scepticism about their ability, training, knowledge, or skills. These clinicians indicated that they were unfamiliar with methods, found research evidence complex, and were unconfident in assessing research evidence independently. For instance, a Family Physician with a fellowship training did not feel to own the skills or knowledge to evaluate research evidence independently:

*“I can’t say I spent a lot of time picking apart how these tools got created and what data it came from. I don’t know that I have the mental capacity… or the knowledge to trust what I think.” (IN63)*

A faculty who taught for decades expressed doubts about residents’ ability to critically appraise cardiovascular CPR studies:

*“By the end of their residency, I might have a few Family Medicine residents who I think are able to judge the quality of a prediction rule. ... I don't think most of them leave, the certainly 90% of my residents, don't leave their training with those skills. They probably could sweat their way through it, but it is not going to be pleasant and the quality of their outcomes.” (GR91)*

A Family Physician, who was also a very experienced researcher, felt it was difficult to read and critically appraise cardiovascular CPR studies:

*“I mean we all were ‘taught.’ But, … even as a researcher and someone who has been trained quite extensively in research methods, I think it's extremely difficult for even someone like myself to critically read an article well, and interpret the results.” (PX33)*

Therefore, the Family Physician argued it was “unrealistic” to think that typical clinicians could critically appraise research evidence about cardiovascular CPRs:

*“I think that... it's unrealistic to think that students, residents, practising providers are actually going to be able to critically appraise the literature and come to their own conclusions... about very much.” (PX33)*

Although counterintuitive, some of these primary care clinicians with thirteen to thirty five years of clinical experience, who are in a position to educate students, residents, and other clinicians, seem to be sceptical about the ability to search, appraise, and use evidence about cardiovascular CPRs.

B. Be acquainted and assume

When clinicians have unfavourable perceptions of or experiences with research, they may not actively seek and evaluate research evidence about cardiovascular CPRs. Even so, clinicians could be passively acquainted with research evidence about cardiovascular CPRs. In this case, they might not make a deliberate judgment about whether they could trust the research evidence. For example, some interview participants, who were unfamiliar with, distrusted, or found it challenging to use research evidence, came in contact with research evidence about cardiovascular CPRs through didactic sessions, rounding, or journal club. Other participants were introduced to research evidence about cardiovascular CPRs via their clinical colleagues, administrative role, and online resources. Here, a Family Physician, who found it challenging to search for and read cardiovascular CPR studies, describes the first encounter with research evidence behind a new cardiovascular CPR:

*“When things changed, … we had a big didactic session about it and a journal club. And, I remember us having a discussion about the research behind it.” (BG35)*

A Physician Assistant who did not have time to look up research evidence because of busy work and family life came to know about cardiovascular CPR studies by reading e-mails:

*“I get like, these e-mails from … Healios … a program that you have subscribed to, and I do some continuing medical education on. … I get tons of ‘Healio Minute’ e-mails, and … I also get e-mails from PA news [and] Doximity.” (TF63)*

Some clinicians, who were unfamiliar with, distrusted, or found it challenging to use research evidence, did not explicitly judge the validity of research evidence. For example, a resident in a General Internal Medicine did not pore over articles and judge the credibility of them because it was difficult to understand the details:

*“It’s not so hard to get into the details that maybe go right over my head. But like, ‘do I really care about all of these little details?’ Yeah. So, I do more abstracts, skim them than … delve into the articles.” (SY54)*

Instead of critically evaluating research evidence, these clinicians assumed others who had more time, resources, and skills had already assessed the evidence. For instance, a Family Physician, who did not feel confident in evaluating research evidence, explained the reason for not reviewing the evidence behind cardiovascular CPRs:

*“I assumed that somebody smarter than me already thought through this. … I assumed that there's smarter people out there that have figured this out, and I don't have to go down this path myself. That's a big assumption.” (IN63)*

Some clinicians, who were unable to critically appraise cardiovascular CPR studies, felt they could not help but trust, have faith in, and go with others’ judgment on the evidence. For example, a family physician, who was unfamiliar with methods used in cardiovascular CPR studies and had difficulty interpreting the results, felt clinicians were led to trust the authors, editors, specialty societies, and guidelines:

*“I think, … in the life of a busy primary care clinician, … we're largely at the mercy of the medical journal editors, … other [specialty] societies, or … guideline writing organizations.” (ZY58)*

Some clinicians, who did not critically evaluate the evidence, assumed that information was credible if it came from certain sources. A busy senior Family Physician, who did not feel experienced with critically appraising cardiovascular CPR studies, described how the credibility of articles was judged:

*“I think it's helpful to go to a journal … like New England Journal or Annals of Internal Medicine. … I think … those papers are pretty tried and true. … If you are asking me, I'll say, ‘well, … let's look at some of the … reputable papers’…” (JA36)*

Similarly, a family physician and researcher who did not critically appraise research evidence determined the credibility of information according to who the authors were.

*“I … sort of look at who are these authors, … you know. Are they people who I think are trustworthy? … I think as a practitioner, I think that there are a lot of leaps of faith. ... I depend on particular authors.” (PX33)*

Therefore, when primary care clinicians do not make any deliberate judgement about research evidence, they assume that someone else had evaluated the evidence, they can go with others’ interpretation of the evidence, or they can trust the evidence from certain sources.

C. Do not rely on evidence

When clinicians have negative perceptions of or experiences with cardiovascular CPR research, they may not rely on research evidence when making decisions about using cardiovascular CPRs. Some participants, who were unfamiliar with, distrusted, or found it challenging to use research evidence, did not seem to rely on research evidence for their decisions about using cardiovascular CPRs. Therefore, research evidence had little influence. For example, a General Internal Medicine resident who frequently used the ACC/AHA pooled cohort equation had not looked up research evidence behind this CPR:

*"I haven’t... done a whole lot of my own digging. … I ... haven’t looked up … the primary research behind it.” (SY54)*

Similarly, a Family Physician explained the reason for not considering research evidence when deciding to use the CHA_2_DS_2_-VASc and HAS-BLED scores [49, 56]:

*“The … statistical analysis involved is not something that probably most primary care clinicians are going to be immediately familiar with. … So I wouldn't say that the… research suggesting that … one tool may or may not be as accurate has affected my choice of the tool.” (ZY58)*

Even when some clinicians were aware of research evidence showing limitations of a cardiovascular CPR, their decisions were not influenced by the evidence. For example, a young Family Physician, who learned about the ACC/AHA pooled cohort equation [51] during residency training, continued to use the CPR despite knowing its inaccuracy:

*“ … the ASCVD (equation), I … learned a lot about … problems with it, like with under predicting or over predicting for non-white people … But I still use it.” (BG35)*

Therefore, even when some clinicians are aware of research evidence, it seems to have a limited role in their decisions about using cardiovascular CPRs.

D. Evidence-unrelated factors

When clinicians do not rely on research evidence to make a decision about their use a cardiovascular CPR, they might depend on factors unrelated to research evidence. Our interview participants relied on six factors unrelated to research evidence: guideline recommendations, others’ use, comfort, intuition, assuming infallibility, and features of cardiovascular CPRs. Many clinicians trusted guideline recommendations and referred to them when selecting cardiovascular CPRs without clearly understanding whether the recommendations were based on the comprehensive evaluation of existing evidence, and therefore are truly the best practices, or not. A resident physician in an Internal Medicine explained the reason for using the CHA_2_DS_2_-VASc score [49, 56]:

*“It's part of the ACCP guidelines. … Sometimes, I just rely on what the guidelines say. … I don't know if I have … a good linear way of determining whether it's a useful tool or not.” (FA84)*

Similarly, a Family Physician who did not look up any studies about the cardiovascular CPR, felt that clinicians had to trust guideline developing organizations and “be on board with” their recommendations:

*“I assumed ... if it's a generally accepted thing, you know, the standard of care, then I should probably be on board with that … if it's endorsed by, you know, like the American Heart Association, or the American College of Cardiology, or the American Academy of Family Physicians. … I kind of have to trust them.” (IN63)*

Some clinicians went along with other people’s decisions. They chose cardiovascular CPRs that were a part of standard practice in their community and were used or suggested by their colleagues, consultants, pharmacists, and supervisors. An experienced Family Physician used cardiovascular CPRs typically expected to be used among medical staff members:

*“These are the ones that are community standard here … they're the ones that the medical staff here is … most familiar with. … if you want to talk to your consultant, those are … the magic words. They'll understand what you're trying to say.” (GR91)*

Other clinicians used cardiovascular CPRs that they felt comfortable because they learned the CPRs in medical school or residency training programme, were aware of and familiar with the CPRs, and were already used to the CPRs. An Internist continued to use the same cardiovascular CPR after completing medical school and residency training, rather than evaluating the advantages and disadvantages of other available CPRs:

*“I've been using it for a long period of time. ... it's become habit. … it's the one that I've chosen and decided to use on a regular basis. ... I was introduced to it. ... during training and throughout residency, medical school clinic clinical rotations. ... I've come across a few others. And, I suspect I just don't use them because I'm familiar with the one that I have used.” (IS78)*

Rather than making a judgement about the evidence behind cardiovascular CPRs, some clinicians assumed the cardiovascular CPRs they used were accurate. A Family Physician who did not verify evidence behind the cardiovascular CPRs due to the lack of ability and time assumed that cardiovascular CPRs were “infallible”:

*“I think … it's common in medicine, … to assume that these things are infallible. You know, the lab tests are perfect, imaging studies are perfect, and prediction … is perfect as it can be. So, I … just kind of assumed that it's as good as we got.” (IN63)*

Some clinicians chose to use cardiovascular CPRs that had sensible, available, and comprehensive predictors, assessed clinically relevant outcomes, were easy to use, and were useful when managing their patients. A Family Physician who worked at a University clinic explained the reason for using the ACC/AHA pooled cohort equation:

*“Our EPIC has a cardiovascular disease risk calculator that's embedded in a dot phrase. … I can put in ‘.ascvd’ when I'm in a patient's chart, and… it will use the data … in the chart to pull up their risk based on the most recent American College of Cardiology risk calculator. … [It] included … a more complete set of predictors … So I … transferred over to it. (PX33)*

These features of cardiovascular CPRs seem to play an important role in primary care clinicians’ decisions about using cardiovascular CPRs when their decisions are not based on research evidence.

## Discussion

### Summary of findings

In this qualitative interview study, we explored how research evidence influenced primary care clinicians’ interactions with and uses of research evidence in their decisions about using cardiovascular CPRs, and identified two main themes: “Seek and judge” and “be acquainted and assume”. Some clinicians were familiar with, trusted, or felt confident in using research evidence about cardiovascular CPRs. These favourable perceptions of and experiences, which were often noted in more junior clinicians who recently completed their training, led some clinicians to actively seave and evaluate the evidence about cardiovascular CPRs. These clinicians appeared to involve research evidence when deciding whether or how to use cardiovascular CPRs. However, rather than depending on study design or internal validity, they often relied on certain features of cardiovascular CPRs studies that might not be the most crucial in ensuring the evidence was high-quality [16, 57, 58], when selecting the CPRs. Furthermore, whether the evidence was applicable to clinicians’ own patients in primary care practices seemed to be a key determinant for selecting their cardiovascular CPRs.

On the other hand, some clinicians were unfamiliar with, distrusted, or found it challenging to use research evidence about cardiovascular CPRs. Surprisingly, these negative perceptions of and experiences with cardiovascular CPR research were often seen in senior clinicians with more clinical, teaching, or research experiences. Instead of actively seeking and judging, these clinicians tended to be passively acquainted with research evidence about cardiovascular CPRs through their training, colleagues, and online resources. These clinicians seemed to assume that someone else had evaluated the evidence, they could go with others’ interpretation of the evidence, or they could trust the evidence if it came from certain sources. Therefore, these clinicians did not rely on research evidence when deciding about using cardiovascular CPRs but referred to guideline recommendations, others’ use, comfort, intuition, assuming infallibility, and features of cardiovascular CPRs.

### Comparison with existing literature

In an ethnographic study of two general practices by Gabbay and May [59], the authors found that general practitioners (GPs) relied on “Mindline” for their clinical decisions but never went through the linear steps of EBM during two years of intermittent observations. Contrary to this, one of the main themes, “seek and judge”, suggests that primary care clinicians might actively search for and evaluate research evidence about cardiovascular CPRs and use the information when making decisions about using the CPRs. Although the theme, “seek and judge”, does not necessarily imply that clinicians are going through the linear processes of EBM, it is possible that the use of research evidence in primary care has progressed since Gabbay and May published their paper in 2004 [59], possibly related to advancements in medical education on how to use evidence in recent years [60–65].

Further, based on the findings that younger, more recently trained clinicians tended to express confidence in using research evidence, it can be hypothesized that advances in the EBM education might have improved clinicians’ perceptions about using research evidence over the years. In fact, evidence shows that physicians’ exposures to EBM education during their training have been increasing. For example, a 1998 national survey of Internal Medicine residency programmes in the USA showed that only 36.8% (99 of 269) offered a dedicated EBM curriculum [66]. Similarly, in a 2000 national survey of Emergency Medicine residency programmes in the USA, although 81.5% (53 of 65) of residency programmes included some degree of EBM teaching, 66.2% (43 of 65) spent less than five hours annually on EBM education [67]. On the other hand, 84.6% (11 of 13) of Canadian Emergency Medicine residency programmes participated in a 2010 national survey offered formal EBM curricula, 61.5% (8 of 11) spent at least ten or more sessions annually, and 53.8% (7 of 13) had online EBM resources for education [68].

We also found that more senior clinicians with more clinical, research, or teaching experiences who might be in a position to teach EBM tended to be less confident about the ability to search for, appraise, and use research evidence about cardiovascular CPRs. Existing literature also consistently shows that a lack of appropriately trained enthusiastic faculties is one of the biggest barriers to EBM education [67–70]. For example, in a 1999 national survey of Internal Medicine clerkship directors in the USA, inadequately trained faculties (82.6%, 90 of 109) and a lack of faculty enthusiasm (43.1%, 47 of 109) were identified as some of the key barriers for EBM education [69].

One of the most important evidence-related factors for clinicians’ decisions about using cardiovascular CPRs in our study was their understanding from research evidence that the CPRs were applicable to clinicians’ own patients. Similarly, a survey and focus group study found that GPs distrusted the accuracy of cardiovascular CPRs in their specific patient populations because these CPRs were derived from different populations [37].

We found that clinicians’ decisions about using cardiovascular CPRs were influenced by several factors unrelated to research evidence. One of these evidence-unrelated factors was the features of cardiovascular CPRs. Many existing studies also identified CPR related features, such as whether CPRs included adequate predictors, evaluated relevant outcomes, were easy to use, or integrated into electronic health records (EHRs), as the key factors that influenced the use of CPRs [32–36, 39, 71–78]. The other evidence-unrelated factor that influenced the clinicians’ decisions about using cardiovascular CPRs in our study was their intuition that using CPRs improved care. Similarly, several existing studies also showed that using CPRs is promoted when users perceive CPRs as clinically helpful [32, 35, 77–81]. Also, previous studies found that clinicians’ knowledge of [33, 73, 82] and familiarity with [77, 79, 81] CPRs influenced the use of CPRs, which is consistent with our study’s finding that clinicians used cardiovascular CPRs they felt comfortable with or were already accustomed to. Lastly, in an internet survey of Emergency Physicians, 78.0% of participants indicated that they were “more likely to use a CPR if it was used regularly by their colleagues” [35], which is also in line with our findings that clinicians used cardiovascular CPRs that were a part of standard practice in their community and were used by their colleagues.

### Strengths and Limitations

We purposively selected a maximum variation sample of 15 primary care clinicians in regards to factors that might determine perceptions and experiences with cardiovascular CPR research. Hence, we were able to identify two common themes among clinicians with a wide range of use of cardiovascular CPRs, research experience, and consideration of research evidence in decisions about using cardiovascular CPRs. However, because the participants were recruited from the WWAMI region Practice and Research Network (WPRN), it is possible that clinicians with interests in research were overrepresented; 33.3% of our participants had some research experience.

Because there was little prior knowledge about this topic, we used an inductive approach to generate hypotheses about how clinicians’ perceptions of and experiences with cardiovascular CPR research influence their decisions about using cardiovascular CPRs. However, we did not test the credibility of these hypotheses. Further, although there is no reason to suspect that our study's findings will not apply to CPRs in other clinical domains, our findings' transferability should be confirmed in a new study.

We did not to carry out respondent validation in our study for the following reasons. Firstly, respondent validation in qualitative research is still a somewhat debated topic [83, 84] and some have questioned the value of this technique as a tool to increase credibility [85, 86]. Secondly, there are a number of practical issues such as reliance on participants memory [83], inability for participants to truly validate other participants’ views and experiences [87], and possibility that the views of participants could have evolved [88]. Lastly, respondent validation can be useful when it aligns with research design and is conducted purposefully and transparently. Because our study was rather pragmatic and descriptive in nature, we concluded that respondent validation was less of a concern.

### Research and clinical implications

A systematic review showed that teaching EBM to trainees improved knowledge about EBM but did not change attitude or behaviour [89]. One of the reasons that the improvement is limited to knowledge might be because the educational efforts to promote EBM have been mainly focused on knowledge and skills of EBM steps [66, 90]. Further, existing evidence about the effectiveness of educational interventions to promote EBM primarily evaluated the impact on knowledge and skills rather than on attitude, confidence, and behaviour [91, 92]. Contrary to these practices in current efforts to promote EBM, we found that clinicians’ familiarity, trust, and confidence might play an important role in their encounter, evaluation, and use of research evidence. Therefore, researchers designing interventions to promote the utilization of high-quality evidence in clinical decisions should consider expanding their focus from improving clinicians’ knowledge and skills to increasing their familiarity, trust, and confidence with research evidence.

All stakeholders involved in generating research evidence, including researchers, journal editors, and funders, should understand that clinicians’ unfamiliarity, distrust, and challenges regarding the use of research evidence might hinder them from using research evidence in decision making. Therefore, these stakeholders of research should ensure that the evidence generated is not only high-quality but also easy for clinicians to access, read and understand, and use in their clinical decisions.

Those clinicians who are already familiar with, trust, and confident in using research evidence might be able to maintain, promote, and reinforce their familiarity, trust, and confidence to use research evidence by continuing to seek, judge, and use research evidence in their decisions. However, understanding that primary care clinicians often depend on various factors unrelated to research evidence when making clinical decisions, guideline developers and policymakers should systematically evaluate existing evidence and carefully base their recommendations on the best available evidence.

## Conclusion

CPRs are designed to be used by clinicians and patients to make evidence-informed healthcare decisions. Evidence from cardiovascular CPR research cannot be valuable unless it is utilised by clinicians, patients, or policymakers to support the use of cardiovascular CPRs. In this study, we found that primary care clinicians’ perceptions of and experiences with cardiovascular CPR research might influence the use of research evidence in this process. Therefore, future efforts to promote the use of research evidence in decisions about cardiovascular CPRs should consider targeting and evaluating clinicians’ familiarity, trust, and confidence, in addition to knowledge and skills to use evidence. At the same time, it is important to recognize clinicians’ unfamiliarity, distrust, and challenges regarding the use of evidence as key barriers in evidence-based decision-making, which might not be easily resolved. Because these clinicians often rely on factors other than research evidence such as guideline recommendations, stakeholders of research such as guideline developers and policymakers should ensure that their recommendations are based on the best available knowledge from systematically reviewing relevant existing evidence.

### Supplementary Information

**Additional file 1.** Search strategy to identify studies that evaluated the influence of research evidence on the uptake of clinical prediction rules.

**Additional file 2.** Interview topic guide.

## Data Availability

The datasets used and/or analysed during the current study are available from the corresponding author on reasonable request. We opted not to deposit the data collected from this study in a publically available source because of potentially sensitive nature of information included in the data.

## Abbreviations

CPR: Clinical prediction rule
EBM: Evidence-based medicine
WPRN: WWAMI region Practice and Research Network
WWAMI: Washington, Wyoming, Alaska, Montana and Idaho
MS IDREC: Medical Sciences Interdivisional Research Ethics Committee
HRT: Hormone replacement therapy
GP: General practitioner
HER: Electronic health record
