# Supplementary material for: Influence of research evidence on the use of cardiovascular clinical prediction rules in primary care: an exploratory qualitative interview study
Source: BMC Prim Care. 2023 Sep 20;24:194. doi: 10.1186/s12875-023-02155-w (PMC10512575; doi:10.1186/s12875-023-02155-w)
Supplement: Supplementary file 1 — Additional file 1. Search strategy to identify studies that evaluated the influence of research evidence on the uptake of clinical prediction rules. [file 12875_2023_2155_MOESM1_ESM.docx]

## **Additional file 1** Search strategy to identify studies that evaluated the influence of research evidence on the uptake of clinical prediction rules.

1. Forecasting/

2. Models, Statistical/

3. Prognosis/

4. Multivariate Analysis/

5. 1 or 2 or 3 or 4

6. (Adopt$ or Use$ or Usage or Using or Uptake or Utilise $ or Aware$ or Knowledge$ or Familiar$).ti.

7. Barrier$.mp.

8. Facilitator$.mp.

9. "Attitude of Health Personnel"/

10. 7 or 8 or 9

11. 5 and 6 and 10
