# Supplementary material for: Influence of research evidence on the use of cardiovascular clinical prediction rules in primary care: an exploratory qualitative interview study
Source: BMC Prim Care. 2023 Sep 20;24:194. doi: 10.1186/s12875-023-02155-w (PMC10512575; doi:10.1186/s12875-023-02155-w)
Supplement: Supplementary file 2 — Additional file 2. Interview topic guide. [file 12875_2023_2155_MOESM2_ESM.docx]

**Additional file 2** Interview topic guide.

Topic guide for interviews with primary care providers (PCPs)

| Research question/topic | Key concept | Interview question | Probe/follow-up question |
| --- | --- | --- | --- |
| Introduction | 1. Introduction and context | Greeting and introduction.  Briefly go over the interview purpose, scope, and process.  Do you have any questions about the interview? Could you please confirm giving me permission to interview you and audio-recording the interview? | Confirm written consent.  Obtain verbal consent when needed. |
| Transition | 1. Clinical prediction rule (CPR) use | Could you please start by briefly telling me about you and your practice?  In the online questionnaire, you indicated that you use cardiovascular CPRs rarely/occasionally/in most or all relevant cases. Can you tell me which CPRs you use and how you use them? | Ask a small follow-up question to build up a rapport.  There are many similar CPRs for the problem. How did you decide that the CPR was the one to use in your practice?  Are there any guideline-recommended cardiovascular CPRs that you are not using? Why? |
| What are PCPs’ perceptions of and experiences with cardiovascular CPR research? | 1. Familiarity and perception of cardiovascular CPR research | What does it mean to you when you hear the term “cardiovascular CPR research”? How would you define or describe it?  You also indicated that you considered research evidence before deciding to use the CPR in the online questionnaire. What specific research evidence did you consider?  How did you know that using this CPR will accurately predict the risk of your patients?  How did you know that using it will improve your practice? |  |
|  | 1. Experience of using cardiovascular CPR research | If you recall reading any research paper about cardiovascular CPR, can you tell me about your experience with the paper?  How did you come across it? How easy was it to read and understand the paper?  How did you determine that the results of the paper were trustworthy? | - What were the challenges? |
|  | 1. Views on cardiovascular CPR research | What are your thoughts/views on cardiovascular CPR research?  Do you think PCPs should consider research evidence when he or she decides to use the CPR? Why? |  |
| How do these perceptions and experiences influence their use of cardiovascular CPRs in practice? | 1. Decision making | Did research evidence influence whether and how you use the CPR?  How did these your experiences and thoughts on CPR research influence your decision to use the CPR? | - What are the important things that will make you choose (or not choose) certain CPRs? |
| Closing |  | Thank the PCP for participating. |  |
